# MicroRNA let-7g and let-7i inhibit hepatoma cell growth concurrently via downregulation of the anti-apoptotic protein B-cell lymphoma-extra large

**DOI:** 10.3892/ol.2014.2706

**Published:** 2014-11-12

**Authors:** LINGJIAO WU, QIANGFENG WANG, JIAN YAO, HAN JIANG, CHENG XIAO, FUSHENG WU

**Affiliations:** 1 State Key Laboratory for Diagnosis and Treatment of Infectious Diseases, The First Affiliated Hospital, Zhejiang University, Hangzhou, Zhejiang 310003, P.R. China; 2 Department of Surgical Oncology, The First Affiliated Hospital, Zhejiang University, Hangzhou, Zhejiang 310003, P.R. China

**Keywords:** miR-let-7g/i, hepatocellular carcinoma, apoptosis, anti-apoptotic protein, coordinately

## Abstract

Let-7 family members have been identified as tumor-suppressing microRNAs, which are important in human hepatocellular carcinoma (HCC). These family members may function differently as a result of different base sequences at the 3′end. The aim of this study was to determine the antitumor effects of miR-let-7g/i (let-7g/i) on HCC cells and to investigate whether let-7g and let-7i have a combinatorial effect on HCC. The expression levels of let-7g/i in hepatoma cells were determined by quantitative reverse transcription polymerase chain reaction. In addition, a 5-ethynyl-2′-deoxyuridine retention assay and flow cytometry analysis were used to detect the effect of let-7g/i on the proliferation and apoptosis of BEL-7402 cells, respectively. The expression of anti-apoptotic protein B-cell lymphoma-extra large (Bcl-xL) was analyzed using western blot analysis. The results revealed that the expression levels of let-7g/i were significantly decreased in HCC cell lines when compared with L-02 cells. Furthermore, the overexpression of let-7g/i significantly suppressed DNA replication, inhibited cell proliferation and promoted apoptosis of BEL-7402 hepatoma cells. The expression of the anti-apoptotic protein, Bcl-xL, was inhibited by the combined role of let-7g and let-7i. We hypothesize that let-7g and let-7i exhibit a concurrent effect to regulate cell proliferation and the apoptosis of hepatoma cells, and this function is mediated by the Bcl-xL protein.

## Introduction

**Figure 1:**
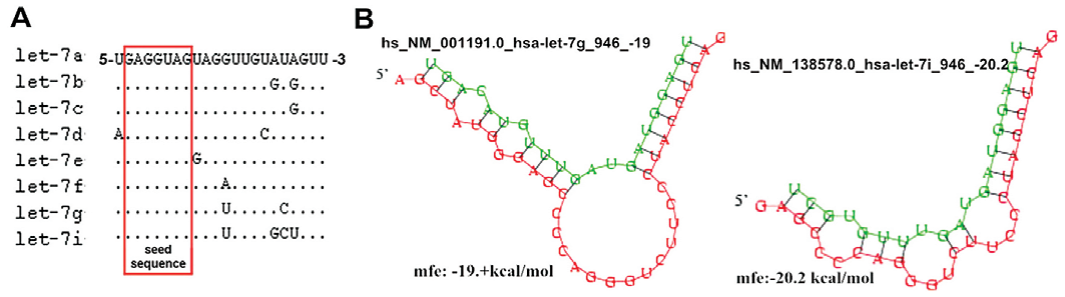
(A) Differences between the base sequences of the let-7 family members. The 2–8 highly conserved base area is known as the seed sequence. (B) The binding mode of let-7g/7i with Bcl-xL was predicted by the bioinformatics of PICTAR (http://www.pictar.org/).

MicroRNAs (miRNAs), which are small, non-coding RNAs that are 18–25 nucleotides in length, are involved in gene regulation. miRNAs bind to the 3′untranslated region (3′UTR) of target mRNAs to inhibit translation or induce the degradation of mRNA (1–3). A previous study revealed that ~50% of human miRNAs are located at fragile sites of the genome, which are associated with cancer (4). This indicates that miRNAs may be crucial for cancer progression (1,5). Full-scale analysis of miRNomes has indicated that only 0.9% of miRNAs are expressed abundantly in the normal human liver; however, these miRNAs account for 88.2% of all miRNAs in the liver, and four of the first nine miRNAs belong to the let-7 family (6). Furthermore, let-7 family members have been found to be downregulated in a number of human cancers, including lung, colon, ovarian, uterine leiomyoma and breast cancer, as well as hepatocellular carcinoma (HCC) (7–13), indicating that let-7 miRNAs may present potential tumor suppressors. It has been reported that let-7c induces apoptosis and inhibits HCC cell proliferation *in vitro* (11). In humans, 12 genomic loci have been identified, which encode the let-7 family members, including let-7a-1, -2, -3, let-7b, let-7c, let-7d, let-7e, let-7f-1, -2, let-7g, let-7i and miR-98 (3). These members share the same nucleotide sequence between the second and eight consecutive nucleotides at the 5′end, which are termed ‘seed sequences’, that determine their target genes (Fig. 1A; miRBase v.18.0; http://www.mirbase.org/search.shtml) (14); therefore, it has been hypothesized that each member exhibits the same role. However, the difference in nucleotides at the 3′end indicates that let-7 families are not functionally equivalent with regard to combination strength and efficiency of interactions with target genes (15,16). It has been reported that members of the let-7 family, mir-48, mir-84 and mir-241, which have the same seed sequences but different nucleotide sequences at the 3′end, exhibit specific and redundant roles in the regulation of developmental timing in *Caenorhabditis elegans* (16). An additional study has also indicated that the degree of complementarity between the 3′end of the miRNA and target gene is the structural basis of the same family miRNAs exerting different functions (15).

Bioinformatics analysis (DIANA Lab and PICTAR) (11) indicates that the anti-apoptotic protein B-cell lymphoma-extra large (Bcl-xL) is a target gene of let-7g and let-7i. The aim of the present study was to investigate whether the let-7 family members exhibit specific and/or overlapping roles in human HCC as observed in *Caenorhabditis elegans*. Thus, the antitumor effect of let-7g/i on HCC was investigated, and whether let-7g and let-7i exhibit a concurrent effect on HCC was determined. Furthermore, the effect of let-7g/i on the Bcl-xL protein in HCC cells was analyzed.

## Materials and methods

### Cell culture

The human L-02 liver cell line and hepatoma cell lines, SMMC-7721 and Bel-7402, were purchased from Shanghai Institutes for Biological Sciences of Chinese Academy of Sciences (Shanghai, China). The L-02 and SMMC-7721 cell lines were cultured in endotoxin-free Dulbecco’s modified Eagle’s medium with 10% fetal bovine serum (Gibco Life Technologies, Carlsbad, CA, USA). The BEL-7402 cell line was cultured in RPMI 1640 medium with 10% (vol/vol) fetal bovine serum (Gibco Life Technologies). The cell lines were incubated at 37°C in an atmosphere of 5% CO_2_.

### Transfection

The miR-let-7g/i (let-7g/i) agomir (an engineered miRNA mimic) and a negative control (similar to agomir-let-7g/i but with a scramble seeding sequence) were obtained from Guangzhou RiboBio Co., Ltd., (Guangzhou, China). Cells were plated at 45–50% confluence. The let-7g/i agomir and/or let-7 agomir negative control were transfected into the human hepatoma BEL-7402 cell line using transfection reagent Lipofectamine™ 2000 in Opti-MEM (Gibco Life Technologies), according to the manufacturer’s instructions. One negative control group, which was transfected with 100 nm negative control agomir, and three experimental groups, which were transfected with 100 nm let-7g agomir, 100 nm let-7i agomir or co-transfected with 50 nm let-7g and 50 nm let-7i agomir (let-7g + let-7i) were used. The expression levels of let-7g and let-7i were quantified using the SYBR Premix Ex TaqTM II (Perfect Real Time) kit (Takara Bio, Inc., Otsu, Japan), 24 h after transfection. Briefly, 20 μl PCR reaction mixture was pre-heated at 95°C for 30 sec, followed by 40 cycles at 95°C for 5 sec and 60°C for 34 sec.

### 5-ethynyl-2′-deoxyuridine (EdU) retention assay

An EdU assay was performed using the Cell Light EdU DNA imaging kit (Guangzhou RiboBio Co., Ltd.)to measure the effects of let-7g/i on cellular proliferation. The EdU assay was performed 48 h after cells were transfected with let-7g/i agomir. Cells were seeded in 96-well plates and exposed to 25 mm EdU for 2 h at 37°C, and were then fixed in 4% paraformaldehyde. Following permeabilization with 0.5% Triton X-100 (Amresco LLC, Solon, OH, USA), the 16 Apollo reaction cocktail (Guangzhou RiboBio Co., Ltd.) was added and the cells were incubated for 30 min. Subsequently, the DNA of the cells was stained with Hoechst 33342 (Sigma-Aldrich, St. Louis, MO, USA) for 30 min and visualized under a fluorescent microscope (IX81; Olympus Corporation, Tokyo, Japan). The cell count was analyzed by Image-Pro Plus 6.0 software (Media Cybernetics, Inc., Rockville, MD, USA).

### Cell apoptosis assay

Apoptosis assay was performed with Annexin V-fluorescein isothiocyanate Apoptosis Detection Kit I (BD Pharmingen, San Diego, CA, USA) 48 h following transfection, according to the manufacturer’s instructions. The cell suspension (100 μl) was incubated with 5 μl Annexin V and 5 μl propidium iodide (BD Pharmingen) at room temperature for 10 min. Finally, 400 μl binding buffer was added to each tube and the cells were suspended. The treated cells were analyzed by fluorescence-activated cell sorting using a BD LSR II flow cytometry kit (BD Pharmingen).

### Real-time PCR

Total RNA was isolated from cells using the mirVana miRNA isolation kit (Ambion Life Technologies, Carlsbad, CA, USA) according to the manufacturer’s instructions. A total of 10 ng total RNA was reversely transcribed using the TaqMan MicroRNA Reverse Transcription kit (Applied Biosystems, Foster City, CA, USA). Quantitative PCR was analyzed using SYBR Premix Ex Taq^TM^ II and the ViiA7 real-time PCR system (Applied Biosystems). U6 small nuclear RNA (snRNA) was used to normalize let-7g/7i expression levels. Primers for let-7g (cat no. miRQ0000414-1-1)/7i (cat no. miRQ0000415-1-1) and U6 (cat no. MQP-0201) snRNA were purchased from RiboBio (Guangzhou, China).

### Western blot analysis

Total protein was isolated from cells using cell lysis buffer (Cell Signaling Technology, Inc.) after transfection for 48 h. The protein levels were quantified using a DC Protein Assay (Bio-Rad Laboratories, Hercules, CA, USA). Protein samples (30 μg) were loaded on a 12% SDS-PAGE gels and electroblotted to Immun-Blot polyvinylidene fluoride membranes (Millipore, Billerica, MA, USA). Membranes were blocked and probed with a monoclonal rabbit anti-human Bcl-xL antibody (1:1,000; Epitomics Inc, Burlingame, CA, USA), then washed with Tris-buffered saline and Tween 20 (50 mM Tris, 150 mM NaCl, 0.1% Tween-20; pH 7.6; Sigma-Aldrich), and incubated with a secondary horseradish peroxidase-conjugated goat anti-rabbit antibody (1:5,000; Hangzhou Hua’an Biotechnology Co., Ltd., Hangzhou, China). Protein levels were normalized to total glyceraldehyde 3-phosphate dehydrogenase (GAPDH) using a mouse anti-human GAPDH antibody (1:1,000; Abcam, Cambridge, UK). The intensity of each protein band was quantified by Quantity One version 4.62 software (Bio-Rad Laboratories).

### miRNA target predictions

The algorithms DIANA Lab (http://diana.cslab.ece.ntua.gr/), Pictar (http://pictar.mdc-berlin.de/), and TargetScan (http://www.targetscan.org/) were used to predict let-7 family members that could potentially bind to Bcl-xL mRNA.

### Statistical analysis

Data are presented as the mean ± standard deviation of four independent experiments. Statistical analyses were performed using Microsoft Excel and SPSS software, version 16.0 (SPSS Inc., Chicago, IL, USA). Quantitative PCR data were analyzed as follows: U6 snRNA was used to normalize let-7g/i expression levels. Let-7g/i expression levels were measured using the threshold cycle (Ct), and the fold-change in expression was calculated as 2^−ΔΔCt^. The relative expression of let-7g/i in hematoma cell lines was calculated using the following formula: ΔΔCt = (Ctlet-7g/i − CtU6) cancer − (Ctlet-7g/i − CtU6) L-02. The relative expression of let-7g/i after transfection was calculated using the equation: ΔΔCt = (Ctlet-7i/g − CtU6) post-transfection − (Ctlet-7g/i − CtU6) pro-transfection. Factorial analysis of variance was used to analyze the interaction between let-7g and let-7i in the EdU retention assay and cell apoptosis assay. P<0.05 was considered to indicate a statistically significant difference.

## Results

### let-7g, let-7i and Bcl-xL expression in hepatoma cells

**Figure 2:**
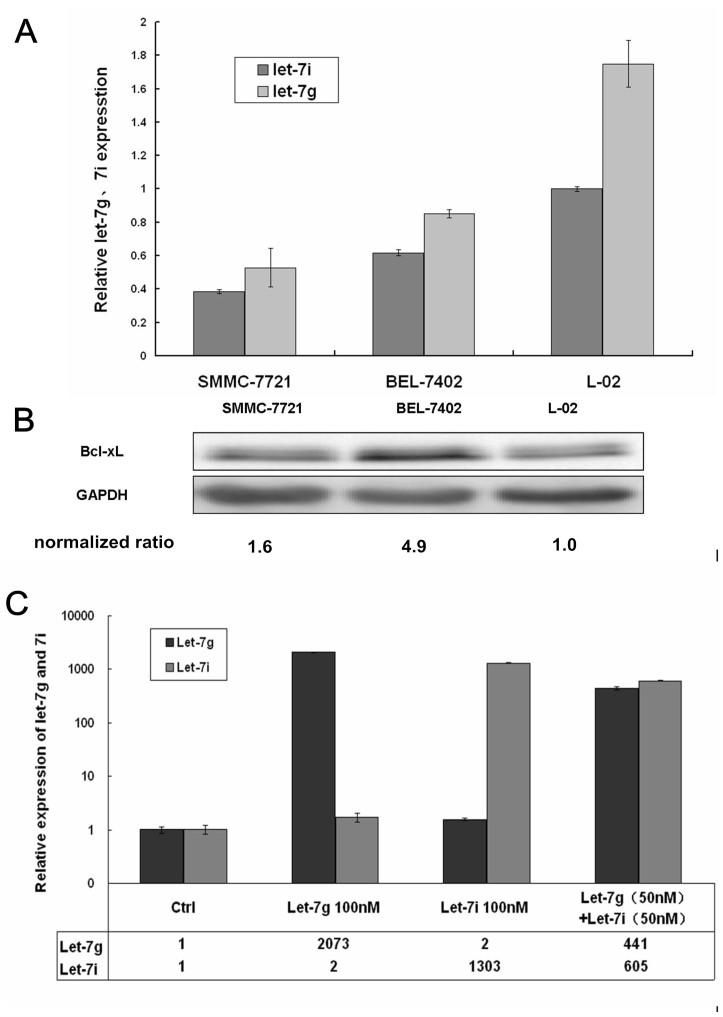
(A) Endogenous expression levels of let-7g/i in SMMC-7721, Bel-7402 and L-02 cell lines were quantified by real-time polymerase chain reaction. (B) The target gene Bcl-xL of let-7g/i in SMMC-7721, Bel-7402 and L-02 cell lines was quantified using western blot analysis. The results (normalized ratio) were standardized against the levels of GAPDH and then normalized to the relative expression level of Bcl-xL in the L-02. (C) The expression levels of let-7g and 7i in BEL-7402 cells transfected with let-7g/i agomir or negative control. let-7g/i agomir was transfected into BEL-7402 cells to increase the expression of let-7g/i. The experiment was divided into four groups: cells transfected with 100 nm negative control agomir (Ctrl), cells transfected with 100 nm let-7g agomir (let-7g 100 nm), cells transfected with 100 nm let-7i agomir (let-7i 100 nm), and cells transfected with 50 nm let-7g and 50 nm let-7i [let-7g (50 nm) + let-7i (50nm)]. miR, microRNA.

let-7g/i expression was decreased in SMMC-7721 and BEL-7402 hepatoma cells when compared with the immortalized liver cell L-02 line (P<0.01) (Fig. 2A).

Bioinformatics analysis (DIANA Lab and PICTAR) predicted that the anti-apoptotic protein Bcl-xL is a target gene of let-7g and let-7i, with a potential binding site on the 3′UTR of Bcl-xL (Fig. 1B). In addition, a previous study demonstrated that Bcl-xL is the direct target of let-7c and -7g in Huh7 hepatoma cells (11). In this study, Bcl-xL protein expression was detected by western blot analysis and it was found that the protein expression of Bcl-xL was increased in the two hepatoma cell lines, when compared with the L-02 cell line (P<0.01) (Fig. 2B).

### Expression levels of let-7g and let-7i in the BEL-7402 cell line following transfection

The fold-changes in let-7g/i levels in the HCC BEL-7402 cell line were detected following transfection with agomirs or the negative control. The expression levels of let-7g in the groups transfected with let-7g or co-transfected with let-7g and let-7i were 2,073- and 441-fold those of the control group, respectively. The expression levels of let-7i in the groups transfected with let-7i and co-transfected with let-7g and let-7i were 1,303- and 605-fold those of the control group, respectively (Fig. 2C).

### Overexpression of let-7g/i inhibits hepatoma cell proliferation

**Figure 3:**
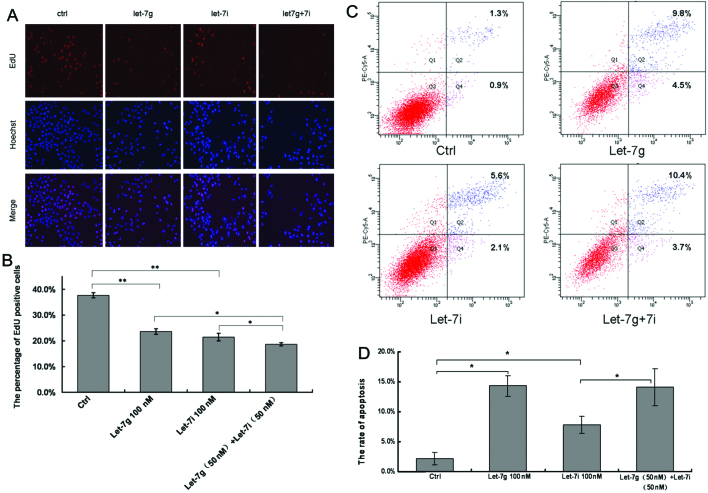
Effects of overexpression of let-7g/i on the proliferation and apoptosis of BEL-7402 cells. (A) The cells were marked by 5-ethynyl-2′-deoxyuridine after transfection for 48 h, then fixed and stained using Apollo and Hoechst 33342. Finally, the cells were observed by fluorescence microscopy and three fluorescent microscope images were captured randomly for each group. (B) Cell count was analyzed by Image-Pro Plus 6.0 software and data analysis was performed using factorial analysis of variance with SPSS software, version 16.0. (C) Cell apoptosis was evaluated using Annexin V-fluorescein isothiocyanate/propidium iodide double staining. Early and late apoptotic cells were combined as Annexin V-positive cells that were used to calculate the percentage of apoptotic cells. (D) Data analysis was performed using factorial analysis of variance with SPSS software, version 16.0. Ctrl, control.

The BEL-7402 cells were transfected with let-7g/i agomir or the negative control to investigate the effects of let-7g/i agomir on HCC cell growth. Hoechst staining nuclei in the experimental groups was dense and contracted (Fig. 3A). DNA replication activity in the groups of transfected with the negative control, let-7g, let-7i and let-7g + 7i was 37.7, 23.6, 21.4 and 18.6%, respectively (Fig. 3B). When compared with the control group, DNA replication activity in the groups transfected with let-7g or let-7i was significantly decreased (P<0.01). When compared with the let-7i group or let-7g group, DNA replication activity of the let-7g + 7i group was inhibited (P<0.05) (Fig. 3B), indicating that there may be a combinatorial effect between let-7g and let-7i.

### Overexpression of let-7g/i induces hepatoma cell apoptosis

BEL-7402 cells were transfected with let-7g/i agomir or the negative control to investigate the effects of let-7g/i on cell apoptosis. The results revealed that transfection with let-7g/i agomir increased the percentage of apoptotic cells in the BEL-7402 cell line (Fig. 3C). The rates of apoptosis in the groups transfected with let-7g, let-7i and let-7g + 7i were 14.3, 7.7 and 14.1%, respectively, which were significantly greater than the rate in the control group (2.2%) (P<0.05; Fig. 3D).

### Co-transfection with Let-7g and let-7i downregulates the expression level of Bcl-xL

**Figure 4:**
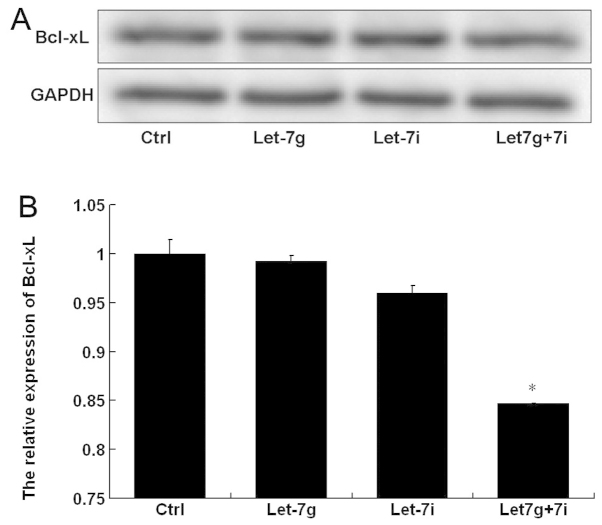
(A) Effects of overexpression of let-7g/i on the Bcl-xL protein. (B) The relative expression of Bcl-xL by gray-value quantitative analysis. Bcl-xL, B-cell lymphoma-extra large; Ctrl, control.

The expression of the anti-apoptotic protein, Bcl-xL, was analyzed by western blot analysis (Fig. 4A). The gray-value quantitative analysis showed that Bcl-xL protein expression in BEL-7402 cells was markedly decreased after co-transfection with let-7g and let-7i compared with that in the control. However, the protein expression was not significantly influenced after transfection with let-7g or let-7i (Fig. 4B), suggesting that let-7g and let-7i exert a combined effect on Bcl-xL protein in BEL-7402 cells.

## Discussion

The correlation between miRNA mutation or altered expression and various human cancers indicates that miRNAs may function as tumor suppressors or oncogenes and, thus, they are referred to as oncomirs (4). It has been demonstrated that the let-7 family are important tumor suppressor genes (17). In this study, the expression of let-7g/i was found to be significantly decreased in HCC cells when compared with the L-02 cell line. These results are consistent with those of Shimizu *et al*, which demonstrated that the expression of let-7b, -7g,-7i, -7d, -7a, -7c and 7e was downregulated in Huh7 cells when compared with normal hepatocytes (11). A similar result was found in the present study, however, different methods and cell lines were used. In addition, the effects of let-7g/i on the biological behavior of hepatoma cells were investigated, and it was found that the overexpression of let-7g/i significantly inhibited the cell proliferation and promoted the cell apoptosis of BEL-7402 cells. miRNAs have been identified as a class of regulatory RNAs in numerous biological processes (18–20). The loss of *let-7* activity induces abnormal development in *Caenorhabditis elegans* (21). In addition, the LIN28/let-7 pathway exhibits a critical pathobiological role in malignant germ cell tumors (22). Furthermore, enforced expression of let-7b inhibited breast cancer cell motility and affected actin dynamics (13).

In this study, co-transfection with let-7g and let-7i was found to exhibit an enhanced effect on BEL-7402 cell proliferation and apoptosis, when compared with the effect of let-7g or let-7i alone. These results indicate that let-7g and let-7i exhibit a combinatorial role in suppressing HCC progression. According to a previous study, the let-7 family members mir-48, mir-84 and mir-241, function together to control the L2-to-L3 transition, likely by base pairing to complementary sites in the hbl-1 3′ UTR, indicating that let-7 family miRNAs function in combination to affect early and late developmental timing decisions (16). Different target genes may be regulated by a single miRNA, and multiple miRNAs may also function together to regulate one or several gene pathways. These regulatory pathways are hypothesized to affect the development of cancer (1,23).

Let-7 miRNAs act as tumor suppressors by modulating major oncogenes, including high mobility group protein (10,24), ras (25) and caspase-3 (26). Bcl-xL is an important member of the anti-apoptotic Bcl-2 family, and has been found to be overexpressed in HCC (27). According to the bioinformatics (DIANA Lab and PICTAR) prediction, Bcl-xL is a direct target gene of let-7, which was also confirmed by a previous molecular study (11). This previous study demonstrated that the overexpression of let-7c or let-7g led to a marked decrease in Bcl-xL expression in Huh7 and HepG2 hepatoma cell lines (11). The present study also showed that Bcl-xL protein expression in the BEL-7402 hepatoma cell line was significantly decreased following combined transfection with let-7g and le-7i. However, the Bcl-xL protein expression was not significantly influenced by transfection with let-7g or let-7i alone. These results indicated that let-7g and let-7i may exhibit a coordinated effect on the Bcl-xL protein. Two different nucleotides have been identified at the 3′end of let-7g and let-7i base sequences, resulting in two different binding sites and modes with Bcl-xL mRNA 3′UTR (Fig. 1B). The two miRNAs augment each other to regulate the Bcl-xL protein. Therefore, the combinatorial role of let-7g and let-7i led to the downregulation of Bcl-xL protein.

In conclusion, let-7g and let-7i exhibit a combined effect to regulate hepatoma cell proliferation and apoptosis, and this function is hypothesized to be mediated via the Bcl-xL protein.

## Abbreviations

miRNA: microRNA
let-7g/i: miRNA-let-7g/i
HCC: hepatocellular carcinoma
UTR: untranslated region
Bcl-xL: B-cell lymphoma-extra large
